# Derangetropy in Probability Distributions and Information Dynamics

**DOI:** 10.3390/e26110996

**Published:** 2024-11-18

**Authors:** Masoud Ataei, Xiaogang Wang

**Affiliations:** 1 Department of Mathematical and Computational Sciences, University of Toronto, Mississauga, ON L5L 1C6, Canada; 2 Department of Mathematics and Statistics, York University, Toronto, ON M3J 1P3, Canada; stevenw@yorku.ca

**Keywords:** information dynamics, probability distributions, functional measures, entropy, combinatorial analysis, differential equations, information theory, equilibrium analysis

## Abstract

We introduce derangetropy, which is a novel functional measure designed to characterize the dynamics of information within probability distributions. Unlike scalar measures such as Shannon entropy, derangetropy offers a functional representation that captures the dispersion of information across the entire support of a distribution. By incorporating self-referential and periodic properties, it provides insights into information dynamics governed by differential equations and equilibrium states. Through combinatorial justifications and empirical analysis, we demonstrate the utility of derangetropy in depicting distribution behavior and evolution, providing a new tool for analyzing complex and hierarchical systems in information theory.

## 1. Introduction

The accurate quantification and analysis of information is critical in many scientific disciplines from data science and information theory to quantum mechanics and statistical physics. Traditionally, Shannon entropy has long served as the foundational measure of information, providing a scalar summary of uncertainty within a probability distribution [1]. While Shannon entropy’s elegance and simplicity have ensured its widespread adoption, it also bears significant limitations, particularly when dealing with complex, non-Gaussian distributions or systems characterized by high-dimensional data.

A key limitation of Shannon entropy is its reduction of the functional space of distributions to a single scalar value, which can lead to identical entropy values for distinct distributions, potentially obscuring their unique characteristics. This issue is particularly pronounced in analyzing non-Gaussian distributions, common in fields such as finance, genomics, and signal processing, where Shannon entropy often fails to capture intricate dependencies [2].

Moreover, Shannon entropy’s reliance on a scalar summary frequently decouples it from other important statistical properties, such as variance, which may offer more direct insights into data spread and reliability. In certain cases, particularly involving nonlinear mappings, Shannon entropy can paradoxically suggest a reduction in information even when measurements enhance the understanding of a variable, as it primarily emphasizes distribution flatness at the expense of other significant features [2,3].

To address these challenges, various extensions of entropy have been proposed, including Rényi entropy and Tsallis entropy [4,5]. While these measures have their merits, they remain fundamentally scalar-centric and do not fully address the need to capture the functional characteristics of information across a distribution’s entire support.

In response to these limitations, we propose *derangetropy*, which is a novel conceptual framework that departs from traditional approaches to information measurement. Unlike existing measures, derangetropy is not merely an extension of entropy but represents a new framework that captures the dynamics of information across the entire support of probability distributions. By incorporating self-referential and periodic properties, derangetropy offers a richer understanding of information dynamics, particularly in systems where information evolves cyclically or through feedback mechanisms such as artificial neural networks.

To contextualize the development of derangetropy within the broader framework of information dynamics, it is helpful to consider recent advances in the study of information flow in complex systems. Tostevin and Ten Wolde examined information transmission in biochemical networks, where they employed mutual information to quantify the flow between input and output trajectories [6]. Their work highlights how biochemical pathways optimize the response to environmental changes, which is a process that is central to the understanding of information dynamics in biological systems.

Extending the analysis of information flows, Horowitz and Esposito explored bipartite models where subsystems exchange information, which is a setup that bridges thermodynamic principles and information theory [7]. Their approach provides a framework for quantifying how information exchange contributes to entropy production in non-equilibrium systems. This bipartite framework was later adapted to quantum systems by Ptaszyński and Esposito, who demonstrated how coherence and entanglement in quantum bipartite systems impact information dynamics [8].

In systems where the environment itself evolves, information dynamics take on additional complexity. Nicoletti and Busiello examined models with dynamically changing environments, demonstrating how adaptability to fluctuating conditions affects information flow [9]. Such studies are particularly relevant for understanding systems that must operate under variable conditions whether in ecological or artificial systems.

The relationship between entropy and information flow is further explored in the work by Ingarden, Kossakowski, and Ohya, which studies open systems where external interactions influence entropy [10]. They provide a foundational perspective on how information flow governs entropy changes, making their work relevant to fields where open-system dynamics play a critical role, such as statistical mechanics and thermodynamics.

These studies collectively underscore the importance of understanding information dynamics across various domains from biochemical networks to quantum systems. In the context of derangetropy, we focus on the distribution of information across the support of probability distributions and introduce informational energies to characterize the interplay between order and disorder within these distributions. This framework extends beyond traditional entropy measures by examining not only how information is quantified but how it is spatially distributed and accumulated within complex systems. By deriving a differential equation that captures the underlying law governing information accumulation, derangetropy has the potential to advance our understanding of how information evolves and adapts across diverse fields, including statistical physics, quantum mechanics, machine learning and biological systems.

We begin in Section 2 by introducing the mathematical definition and key properties of derangetropy. Section 3 analyzes the information dynamics and equilibrium within this framework. In Section 4, we discuss the self-referential and self-similar nature of derangetropy. Section 5 provides a combinatorial perspective on derangetropy, connecting it to fundamental principles of combinatorial analysis. Furthermore, in Section 6, we demonstrate an application of derangetropy to the analysis of biological signals. Finally, Section 7 summarizes the findings and suggests potential avenues for future research.

## 2. Mathematical Definition and Properties

### 2.1. Derangetropy Functional

Let (Ω,F,P) be a probability space, where Ω denotes the sample space, F is a σ-algebra of measurable subsets of Ω, and P is a probability measure on F. Consider a real-valued random variable X:Ω→R that is measurable with respect to the Borel σ-algebra $B_{R}$ on R. Assume that *X* has an absolutely continuous distribution with a *probability density function* (PDF) $f\;\in \;L^{2}\left(R,B_{R},\lambda \right)$, where λ denotes the Lebesgue measure on R. The *cumulative distribution function* (CDF) associated with random variable *X* is given by

$$F\left(x\right)=\int_{-\infty }^{x}f\left(t\right)\;d\lambda \left(t\right),\;x\in R.$$

In the following, we present the formal definition of the derangetropy functional.

**Definition** **1** (Derangetropy). *The derangetropy functional $\rho :L^{2}\left(R,B_{R},\lambda \right)\to L^{2}\left(R,B_{R},\lambda \right)$ is defined by the following mapping*

(1) $$\rho \left[f\right]\left(x\right)=\left(\frac{24}{\pi e}\right)\sin\left(\pi F\left(x\right)\right)F{\left(x\right)}^{F(x)}{\left(1-F\left(x\right)\right)}^{1-F(x)}f\left(x\right),$$

*where f is the PDF associated with random variable X, and ρ[f] refers to the function in $L^{2}\left(R,B_{R},\lambda \right)$ obtained through this mapping. The evaluation of the derangetropy functional at a specific point x∈R is denoted by $\rho_{f}\left(x\right)$.*

The sine function plays a crucial role in modeling systems characterized by cyclical or repetitive phenomena, where information alternates between concentrated and dispersed states. Specifically, the sine function serves as both a *modulation mechanism* and a *projection operator*. As a modulation mechanism, the sine function encodes the cyclical aspects of information content, capturing the periodic nature of information flow in systems with intrinsic cycles, such as those observed in time series data. By modulating the information content, the sine function effectively characterizes the periodic dynamics of information distribution within the probability space. Simultaneously, as a projection operator, the sine function translates the modulated information into a functional space where the dynamics of information flow become more apparent. This dual role facilitates the analysis of complex systems, enabling the representation of higher-dimensional informational structures and their influence on *observable* behavior.

To gain more insights into its structure, the derangetropy functional can be interpreted as a Fourier-type transformation as follows:

(2) $$\rho \left[f\right]\left(x\right)=\left(\frac{24}{\pi e}\right)\sin\left(\pi F\left(x\right)\right)\;e^{-H_{B}\left(F\left(x\right)\right)}f\left(x\right),$$

where

(3) $$H_{B}\left(F\left(x\right)\right)=-F\left(x\right)\log\left(F\left(x\right)\right)-\left(1-F\left(x\right)\right)\log\left(1-F\left(x\right)\right),$$

is the *Shannon entropy* for a Bernoulli distribution with success probability p=F(x). The term $H_{B}\left(F\left(x\right)\right)$ quantifies the uncertainty or informational balance between the regions to the left and right of *x*, as indicated by the CDF values F(x) and 1−F(x), respectively. In this interpretation, the derangetropy functional can be viewed as mapping the current informational energy space, represented by Shannon entropy, into a space characterized by modulation behavior. The exponential term $e^{-H_{B}\left(F\left(x\right)\right)}$ modulates the sine function sin(πF(x)), adding a layer of complexity that reflects how information is concentrated or dispersed across the distribution. This modulation is essential for understanding *localized informational dynamics*, where the oscillations of the sine function mirror the underlying fluctuations in information content.

### 2.2. Key Mathematical Properties

We now establish key mathematical properties of the derangetropy functional that underscore its utility in analyzing probability distributions. For any absolutely continuous PDF f(x), the derangetropy functional ρ[f](x) is a nonlinear operator that belongs to the space $C^{\infty }\left(R\right)$ having the following first derivative

(4) $$\frac{d}{dx}\rho_{f}\left(x\right)=\rho_{f}\left(x\right)\left[\pi \cot\left(\pi F\left(x\right)\right)+\log\left(\frac{F(x)}{1-F(x)}\right)+\frac{f^{\prime }\left(x\right)}{f(x)}\right],$$

where the derivatives are taken with respect to *x*. The following theorem shows that the derangetropy functional involving distribution functions of random variable *X* itself is a valid PDF for another random variable.

**Theorem** **1.** *For any absolutely continuous f(x), the derangetropy functional $\rho_{f}\left(x\right)$ is a valid probability density function.*

**Proof.**  To prove that $\rho_{f}\left(x\right)$ is a valid PDF, we need to show that $\rho_{f}\left(x\right)\geq 0$ for all x∈R and that $\int_{-\infty }^{\infty }\rho_{f}\left(x\right)\;dx=1$. The non-negativity of $\rho_{f}\left(x\right)$ is clear due to the non-negativity of the terms involved in its definition. The normalization condition can further be verified by the change of variables z=F(x), yielding

(5) $$\int_{-\infty }^{\infty }\rho_{f}\left(x\right)\;dx=\int_{0}^{1}\left(\frac{24}{\pi e}\right)\sin\left(\pi z\right)z^{z}{\left(1-z\right)}^{1-z}dz.$$

As shown in Appendix A, the integral

(6) $$\int_{0}^{1}\sin\left(\pi z\right)z^{z}{\left(1-z\right)}^{1-z}dz=\frac{\pi e}{24},$$

which, in turn, implies that

(7) $$\int_{-\infty }^{\infty }\rho_{f}\left(x\right)\;dx=1.$$

Hence, $\rho_{f}\left(x\right)$ is a valid PDF. □

Furthermore, since the derangetropy functional $\rho_{f}\left(x\right)$ is a valid PDF, it possesses well-defined theoretical properties, such as the existence of a mode. The following theorem demonstrates that for any symmetric unimodal distribution, the mode of $\rho_{f}\left(x\right)$ coincides with the median of the underlying distribution.

**Theorem** **2.** *For any symmetric unimodal distribution with an absolutely continuous f(x), the derangetropy functional $\rho_{f}\left(x\right)$ is maximized at the median of the distribution.*

**Proof.**  We aim to demonstrate that $\rho_{f}\left(x\right)$ attains its maximum at the median *m*, where F(m)=0.5. Given the symmetry of the distribution, the median *m* coincides with the mode, and the PDF f(x) is symmetric around *m*, implying that $f^{\prime }\left(m\right)=0$. By analyzing the derivative of the derangetropy functional, it follows that x=m is a critical point of $\rho_{f}\left(x\right)$. To confirm that *m* is the point of maximum, we further examine the second derivative of $\rho_{f}\left(x\right)$ at x=m, which yields

(8) $${\left.\frac{d^{2}\rho_{f}\left(x\right)}{dx^{2}}\right|}_{x=m}=\rho_{f}\left(m\right)\left[-\pi^{2}\csc^{2}\left(\pi F\left(m\right)\right)+{\left.\frac{d^{2}}{dx^{2}}\log\left(\frac{F(x)}{1-F(x)}\right)\right|}_{x=m}+\frac{f^{\prime \prime }\left(m\right)}{f(m)}\right].$$

Since $f^{\prime \prime }\left(m\right)\leq 0$ due to the unimodal nature of the distribution, the second derivative is negative at x=m, confirming that $\rho_{f}\left(x\right)$ has a local maximum at this point. The symmetry of the distribution ensures that this local maximum at *m* is, in fact, the global maximum of the derangetropy functional. Thus, $\rho_{f}\left(x\right)$ is maximized at the median *m* of any symmetric unimodal distribution. □

### 2.3. Empirical Observations and Insights

To illustrate the behavior of the derangetropy functional $\rho_{f}\left(x\right)$, we examine five representative distributions: uniform, normal, exponential, semicircle, and arcsin. These distributions highlight important features such as symmetry, skewness, tail behavior, and boundary effects, offering insights into how probability density functions relate to their corresponding derangetropy functionals.

**Figure 1:**
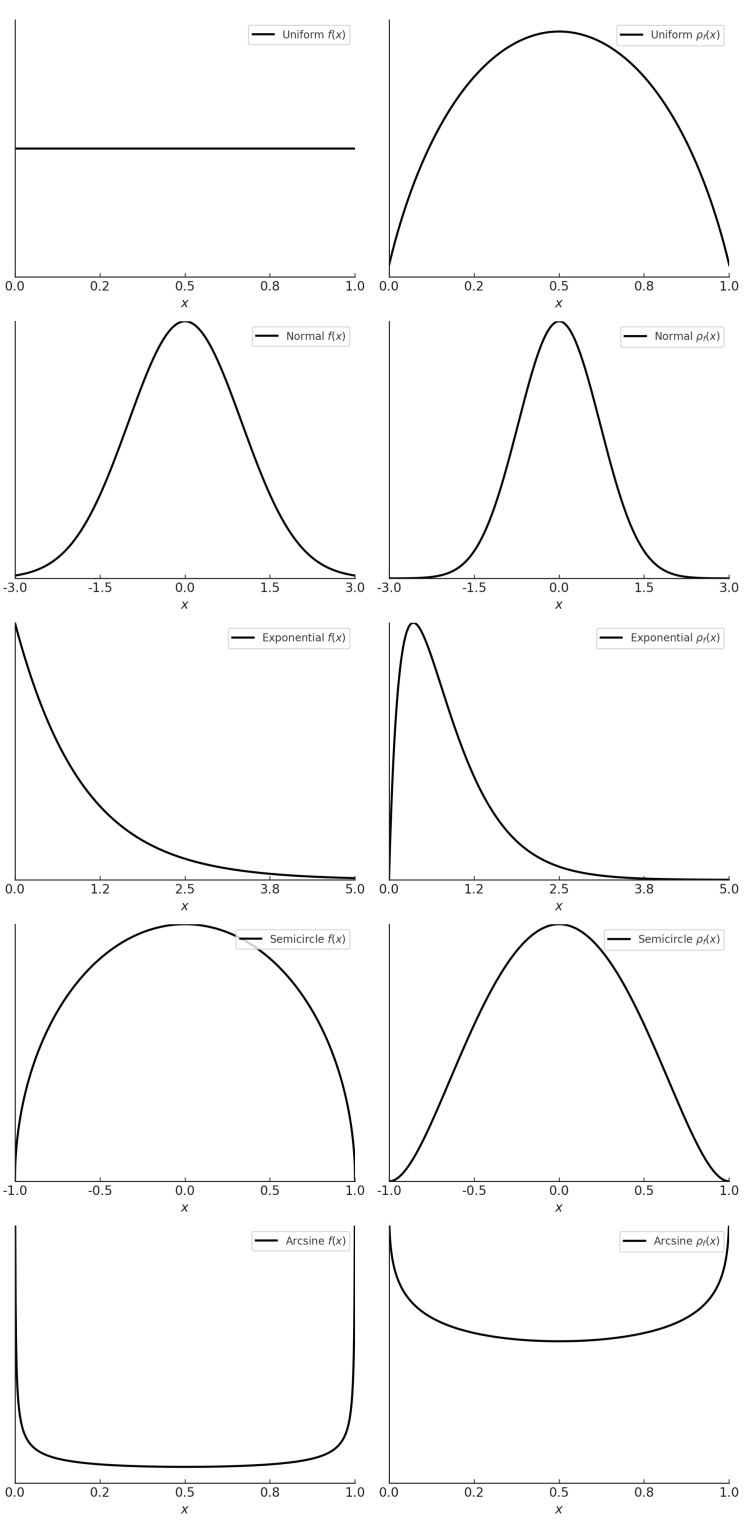
Plots of probability density functions f(x) (**left**) and derangetropy functionals $\rho_{f}\left(x\right)$ (**right**) for uniform(0,1), normal(0,1), exponential(1), semicircle(−1,1), and arcsin(0,1) distributions.

As depicted in Figure 1, for the uniform distribution, f(x) is constant, indicating equal probability for all values in the support. The corresponding derangetropy functional $\rho_{f}\left(x\right)$ forms a semi-parabolic shape, peaking at the center of the distribution, where uncertainty and entropy are highest. As *x* approaches the boundaries, $\rho_{f}\left(x\right)$ decreases smoothly, reflecting a gradual reduction in uncertainty as the system approaches more deterministic regions.

In symmetric distributions such as the normal and semicircle distributions, $\rho_{f}\left(x\right)$ exhibits symmetry with its highest values concentrated where the probability density is greatest. For the normal distribution, $\rho_{f}\left(x\right)$ peaks at the mean (which coincides with the mode and median), capturing the central concentration of probability mass. For the semicircle distribution, which has finite support, $\rho_{f}\left(x\right)$ also peaks near the center, but it decays more gradually toward the boundaries due to the shape of the distribution.

In asymmetric distributions like the exponential distribution, the derangetropy functional reflects the skewness of the distribution. Here, $\rho_{f}\left(x\right)$ rises sharply near the lower boundary, where most of the probability mass is concentrated, leading to an asymmetric shape in $\rho_{f}\left(x\right)$. This sharp rise illustrates how informational content is concentrated in regions where the likelihood of events is highest.

Finally, boundary effects play a significant role in shaping $\rho_{f}\left(x\right)$, particularly in distributions like the arcsin distribution, where $\rho_{f}\left(x\right)$ exhibits pronounced increases near the boundaries, highlighting the sensitivity of informational content to regions of high information concentration. This behavior is particularly important in fields like risk management and extreme value theory.

## 3. Information Dynamics and Equilibrium

### 3.1. Framework for Informational Energy

In this section, we introduce *informational energies* to quantify the distinct aspects of *order* and *disorder* in probabilistic systems. The derangetropy functional reveals how information is distributed, concentrated, or dissipated across the support of a probability distribution, drawing an analogy to energy conservation principles in classical mechanics.

The informational content, described by the derangetropy functional $\rho_{f}\left(x\right)$, can be decomposed as follows:

(9) $$\log\left(\rho_{f}\left(x\right)\right)=\log\left(\sin\left(\pi F\left(x\right)\right)\right)-H_{B}\left(F\left(x\right)\right)+\log\left(f\left(x\right)\right)+C,$$

where $C=\log(\frac{24}{\pi e})$ is a normalization constant. The *total informational energy*, $E_{\mathrm{Total}\;}\left(x\right)$, is defined as

$$E_{\mathrm{Total}\;}\left(x\right)=E_{\mathrm{Modulation}\;}\left(x\right)+E_{\mathrm{Structural}\;}\left(x\right),$$

with *modulation energy* given by

(10) $$E_{\mathrm{Modulation}\;}\left(x\right)=-\log\left(\sin\left(\pi F\left(x\right)\right)\right),$$

and *structural energy* given by

(11) $$E_{\mathrm{Structural}\;}\left(x\right)=H_{B}\left(F\left(x\right)\right)-\log\left(f\left(x\right)\right),$$

where the negative logarithm of derangetropy is referred to as energy in analogy with Boltzmann distributions.

To illustrate these concepts, we consider the uniform distribution on (0,1), where the PDF is f(x)=1 and the CDF is F(x)=x. In this case, the structural energy aligns with Shannon entropy $H_{B}\left(x\right)$, quantifying the system’s intrinsic uncertainty or disorder. Structural energy reaches its maximum at the median (x=0.5), where outcomes are most unpredictable, and it is minimized near the boundaries (x=0 or x=1), where the system becomes highly ordered and predictable. This behavior mirrors thermodynamic entropy, which measures the degree of disorder in physical systems.

By contrast, the modulation energy, represented by −log(sin(πx)), captures the effort required to maintain order in the system. Near the boundaries of the uniform distribution, where the system is most ordered, modulation energy peaks, reflecting the increased energy needed to resist disturbances that could disrupt this order. This represents the work performed to resist disorder and maintain stability. In regions of high uncertainty, such as near x=0.5, less modulation energy is needed because the system is already disordered, and small fluctuations do not significantly affect its overall state.

The total informational energy integrates both structural energy and modulation energy, providing a holistic view of how the system balances disorder and the effort required to stabilize it. In ordered regions, where structural energy is low, modulation energy rises to maintain system stability, compensating for the lack of disorder. Conversely, in disordered regions, where structural energy is high, less modulation energy is required because the system can naturally sustain its fluctuating state. This dynamic mirrors the work–energy relationship in thermodynamics, where total energy reflects both entropy and the energy needed to sustain order. Structural energy thus aligns with entropy, representing disorder, while total informational energy parallels free energy, capturing the system’s balance between disorder and the effort required to maintain order.

**Figure 2:**
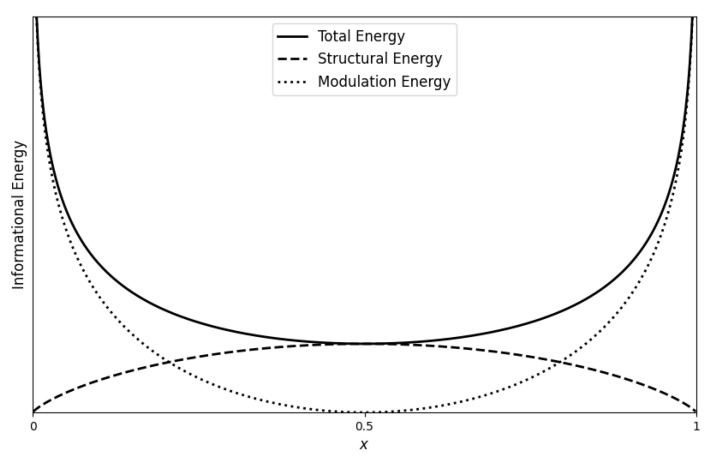
Plots of total (solid line), structural (dashed line) and modulation (dotted line) informational energies for uniform (0, 1) distribution.

The relationship between total informational energy, structural energy, and modulation energy for the uniform distribution is depicted in Figure 2. As shown in this figure, structural energy peaks at the median, reflecting maximum uncertainty, while modulation energy peaks near the boundaries, where the system is most ordered but more sensitive to disturbances. The total energy curve demonstrates that the highest energy expenditure occurs near the boundaries due to the increased effort required to maintain order. In the center of the distribution, where disorder is highest, the total energy is lower because less effort is needed to sustain the naturally fluctuating state.

### 3.2. Equilibrium Analysis

In the derangetropy framework, equilibrium is governed by the interplay between modulation and structural informational energies. Modulation energy measures the system’s resistance to fluctuations, and it is prominent in regions of low probability density, where the system exhibits more order and is sensitive to perturbations. Conversely, structural energy reflects the system’s disorder and uncertainty, which is more pronounced in regions of high probability density. The balance between these two energies elucidates how equilibrium is achieved and maintained across the support of probability distributions.

For any given probability distribution, the total informational energy is the sum of the modulation and structural energy components. In particular, for the uniform distribution on (0,1), the total energy is given by

(12) $$E_{\mathrm{Total}\;}\left(x\right)=-\log\left(\sin\left(\pi x\right)\right)+H_{B}\left(x\right),$$

where −log(sin(πx)) represents modulation energy and $H_{B}\left(x\right)=-x\log\left(x\right)-(1-$x)log(1−x) is the binary entropy function, representing structural energy.

Equilibrium points are identified where the first derivative of total energy with respect to *x* is zero. Differentiating the total energy gives

(13) $$\frac{d}{dx}E_{\mathrm{Total}\;}\left(x\right)=-\pi \cot\left(\pi x\right)+\log\left(\frac{1-x}{x}\right)=-\pi \cot\left(\pi x\right)+2\mathrm{atanh}\left(1-2x\right)\cdot$$

Setting this derivative to zero yields the equilibrium point at x=0.5. Thereafter, the nature of this equilibrium is assessed by examining the second derivative of the total energy given by

(14) $$\frac{d^{2}}{dx^{2}}E_{\mathrm{Total}\;}\left(x\right)=\pi^{2}\csc^{2}\left(\pi x\right)+\frac{1}{x(1-x)}\cdot$$

At x=0.5, the second derivative yields a positive value, i.e.,

(15) $${\left.\frac{d^{2}}{dx^{2}}E_{\mathrm{Total}\;}\left(x\right)\right|}_{x=0.5}=\pi^{2}+4>0,$$

which implies that x=0.5 is a point of minimal energy. In contrast, at the boundaries x=0 and x=1, the total energy increases steeply, indicating instability, since the second derivative of the total energy diverges as *x* approaches these boundaries; i.e.,

(16) $${\left.\frac{d^{2}}{dx^{2}}E_{\mathrm{Total}\;}\left(x\right)\right|}_{x=0\;\mathrm{or}\;x=1}\to \infty .$$

This behavior highlights the instability at the boundaries, where small perturbations can lead to significant deviations from equilibrium. The dominance of modulation energy near these boundaries, coupled with diminishing structural energy, makes these regions highly sensitive to fluctuations.

### 3.3. Differential Equation

The analysis of information dynamics within a probability distribution leads to the derivation of a second-order nonlinear ordinary differential equation that governs the evolution of informational content, as stated in the following theorem.

**Theorem** **3.** *Let X be a random variable following a uniform distribution on the interval (0, 1). Then, the derangetropy functional $\rho_{f}\left(x\right)$ satisfies the following second-order nonlinear ordinary differential equation*

(17) $$\frac{d^{2}\rho_{f}\left(x\right)}{dF{\left(x\right)}^{2}}+4\mathrm{atanh}\left(1-2F\left(x\right)\right)\frac{d\rho_{f}\left(x\right)}{dF(x)}+\left[\pi^{2}-\frac{1}{F(x)(1-F(x))}+4{\mathrm{atanh}}^{2}\left(1-2F\left(x\right)\right)\right]\rho_{f}\left(x\right)=0,$$

*where $\mathrm{atanh}\left(z\right)=\frac{1}{2}\log\left(\frac{1+z}{1-z}\right)$ and the initial conditions are given by*

$${\left.\rho_{f}\left(x\right)\right|}_{F(x)=0}=0,\;\;\mathrm{and}\;{\left.\;\frac{d\rho_{f}\left(x\right)}{dF(x)}\right|}_{F(x)=0}=\frac{24}{e}.$$

**Proof.**  To solve this equation, we first introduce the following integrating factor

(18) $$\begin{array}{cc} \mu (F(x)) & =e^{\int -2\mathrm{atanh}(1-2F(x))dF(x)} \end{array}$$

(19) $$\begin{array}{cc} \; & =\left(F\left(x\right)-1\right)e^{-2F(x)\mathrm{atanh}(1-2F(x))} \end{array}$$

(20) $$\begin{array}{cc} \; & =-F{\left(x\right)}^{F(x)}{\left(1-F\left(x\right)\right)}^{1-F(x)}, \end{array}$$

which, in turn, simplifies the system by eliminating the first-order derivative term. By multiplying the entire equation by this integrating factor and introducing a new function ν(F(x)) such that

(21) $$\rho_{f}\left(x\right)=\mu \left(F\left(x\right)\right)\nu \left(F\left(x\right)\right),$$

we reduce the equation to

(22) $$\frac{d^{2}\nu \left(F\left(x\right)\right)}{dF{\left(x\right)}^{2}}+\pi^{2}\nu \left(F\left(x\right)\right)=0.$$

The general solution to this simplified differential equation is

(23) $$v\left(F\left(x\right)\right)=C_{1}\sin\left(\pi F\left(x\right)\right)+C_{2}\cos\left(\pi F\left(x\right)\right)$$

where $C_{1}$ and $C_{2}$ are constants. Finally, evaluating the initial values yields $C_{1}=\frac{24}{\pi e}$ and $C_{2}=0$, implying that $\rho_{f}\left(x\right)$ is indeed a solution to the differential equation. This solution describes the evolution of the derangetropy functional as a complex interaction of modulation and structural influences across the support of the distribution. □

The behavior of the solution at different points of the distribution reveals key insights into the nature of the informational content. At the median of the distribution, where F(x)=0.5, the term atanh(1−2F(x)) vanishes. This simplifies the equation and leads to a stabilization of the derangetropy functional, indicating that the system is locally stable at this point. The stabilization at the median reflects a state of equilibrium where the competing influences of order and disorder balance each other.

As *x* approaches the boundaries of the distribution, the nonlinear term atanh(1−2F(x)) diverges, introducing significant nonlinearity into the equation. This divergence signals an increased sensitivity of the derangetropy functional near the boundaries. The additional term $\frac{1}{F(x)(1-F(x))}$, which is inversely proportional to the variance of a Bernoulli distribution with success probability p=F(x), further amplifies this boundary sensitivity. As F(x) and 1−F(x) compress their respected probability masses into smaller regions near the boundaries, the differential equation predicts significant changes in the informational content. This heightened sensitivity near the boundaries mirrors the behavior of the modulation energy, which dominates in these regions and makes the system highly responsive to small perturbations.

The interaction between linear and nonlinear dynamics creates a dynamic equilibrium across the distribution. Near the median, the system follows a smoother evolution, reflecting the balance between order and disorder. However, as the system approaches the boundaries, nonlinear effects become increasingly significant, leading to abrupt changes in the rate of evolution of the derangetropy functional. This complex behavior suggests that the derangetropy functional is continuously adapting to the distribution’s local properties.

The differential equation governing the derangetropy functional also bears a strong resemblance to the dynamics of utility theory in economics. As information accumulates within the distribution, the derangetropy functional behaves similarly to economic utility, representing the efficiency with which information is distributed. Initially, the informational content increases rapidly, which is akin to the increasing utility an economic agent derives from the early consumption of a good. This growth continues until the distribution reaches its median, where utility is maximized, and the system achieves a state of optimal balance. The median, therefore, acts as a natural equilibrium point, which is analogous to the point of optimal allocation in utility theory, where resources are most efficiently distributed.

As the system progresses beyond the median and approaches the boundaries, the utility—or the informational content—begins to exhibit diminishing returns. This is similar to the concept of diminishing marginal utility in economics, where the additional benefit gained from consuming more units of a good decreases. In the case of the derangetropy functional, as the distribution approaches its extremes, the rate of change in informational content slows, indicating that an additional concentration of probability mass near the boundaries provides decreasing informational gains. This suggests that over-concentration near the boundaries carries a risk of diminishing efficiency, further reflecting the utility theory analogy.

## 4. Self-Referential Nature

A distinguishing feature of derangetropy is its *self-referential* nature, where $\rho_{f}\left(x\right)$, as a valid PDF, not only encapsulates the informational content of the underlying distribution f(x) but also recursively uncovers the hierarchical structure of its own information content. This recursive relationship is formalized through the following equation

(24) $$\rho_{f}^{\left(n\right)}\left(x\right)=\left(\frac{24}{\pi e}\right)\sin\left(\pi G_{f}^{\left(n-1\right)}\left(x\right)\right){\left(G_{f}^{\left(n-1\right)}\left(x\right)\right)}^{G_{f}^{\left(n-1\right)}\left(x\right)}{\left(1-G_{f}^{\left(n-1\right)}\left(x\right)\right)}^{1-G_{f}^{\left(n-1\right)}\left(x\right)}\rho_{f}^{\left(n-1\right)}\left(x\right),$$

where $\rho_{f}^{\left(n\right)}\left(x\right)$ represents the *n*th iteration of the derangetropy functional, and

$$G_{f}^{\left(n\right)}\left(x\right)=\int_{-\infty }^{x}\rho_{f}^{\left(n\right)}\left(t\right)\;dt$$

is the associated CDF. The initial conditions are set by

$$\rho_{f}^{\left(0\right)}\left(x\right)=f\left(x\right)\;\mathrm{and}\;G_{f}^{\left(0\right)}\left(x\right)=F\left(x\right),$$

where f(x) and F(x) denote the PDF and CDF of the original distribution, respectively. This recursive structure ensures that each subsequent layer $\rho_{f}^{\left(n\right)}\left(x\right)$ integrates the informational content of all preceding layers, constructing a comprehensive hierarchical representation of information.

**Figure 3:**
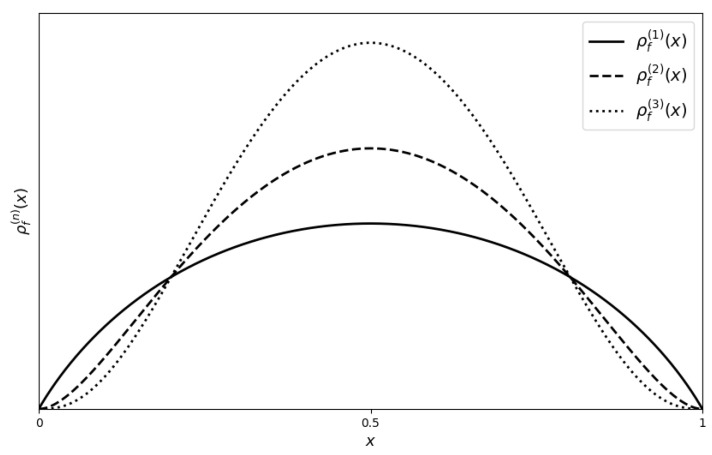
Plots of $\rho_{f}^{\left(1\right)}$ (solid line) and $\rho_{f}^{\left(2\right)}$ (dashed line) for uniform(0,1) distribution.

To elucidate this self-referential property, we consider the first and second layers of the derangetropy functional for the uniform distribution, as illustrated in Figure 3. The first layer, $\rho^{\left(1\right)}\left(x\right)$, reflects the basic informational structure of the uniform distribution. In contrast, the second layer, $\rho^{\left(2\right)}\left(x\right)$, introduces additional complexity, manifesting in pronounced peaks and troughs. This evolution underscores the recursive amplification inherent in the derangetropy functional, progressively unveiling deeper informational layers with each iteration.

**Figure 4:**
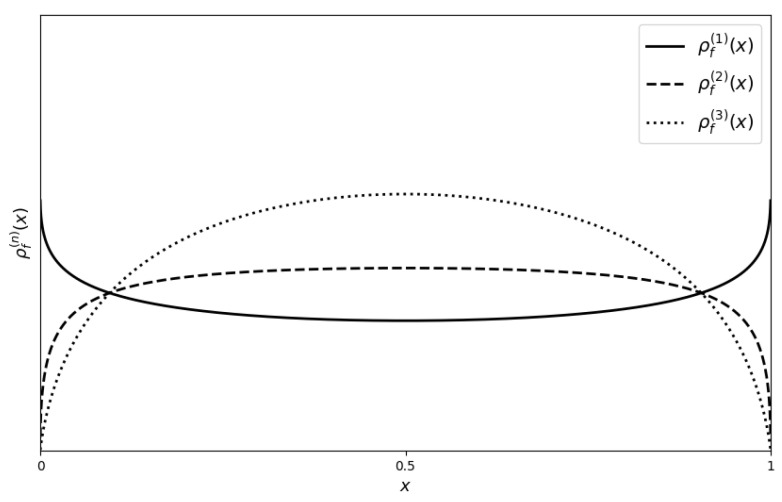
Plots of $\rho_{f}^{\left(1\right)}$ (solid line), $\rho_{f}^{\left(2\right)}$ (dashed line) and $\rho_{f}^{\left(3\right)}$ (dotted line) for Arcsin(−1,1) distribution.

Further insights into the self-referential nature of the derangetropy functional can be obtained by examining its behavior for the arcsin distribution, as shown in Figure 4. The first layer captures the strong boundary effects characteristic of the arcsin distribution, resulting in a pronounced concave upward profile near the interval’s endpoints. As recursion advances to the second layer, these boundary effects are amplified, giving rise to a more complex informational structure. The transition in concavity observed between the first and second layers signifies a shift from a simple, boundary-focused representation to a more intricate depiction of the distribution’s information content. The third layer introduces a semi-parabolic shape, indicative of a stabilization process within the recursive structure, where the previously amplified boundary effects become harmonized. This semi-parabolic curve suggests a tendency toward an informational equilibrium within the distribution as recursion progresses, where initially sharp boundary effects are moderated and the information distribution becomes more balanced.

An intriguing observation emerges when comparing the third-level derangetropy of the arcsin distribution with the first-level derangetropy of the uniform distribution, which closely resemble one another. This resemblance reveals a deep structural connection between these distributions under the framework of the derangetropy functional. More precisely, the transformation $X=\sin^{2}\left(\frac{\pi }{2}U\right)$, where U∼Uniform(0,1), maps the uniform distribution onto the arcsin distribution. In this transformation, the sine function embedded within the derangetropy functional plays an essential role. When applied to the arcsin distribution, which has a CDF given by

(25) $$G_{\mathrm{Arcsin}\;}\left(x\right)=\frac{2}{\pi }\arcsin\left(\sqrt{x}\right),$$

the sine component of the derangetropy simplifies as follows:

(26) $$\sin\left(\pi G_{\arcsin}\left(x\right)\right)=\sin\left(2\arcsin\left(\sqrt{x}\right)\right)=2\sqrt{x(1-x)}.$$

This final expression, $2\sqrt{x(1-x)}$, mirrors the semi-parabolic shape typically observed in the derangetropy functional of a uniform distribution, highlighting a structural similarity between the arcsin and uniform distributions. This connection illustrates how the recursive nature of the derangetropy functional can transform a distribution like the arcsin, which exhibits strong boundary effects, toward a more balanced informational profile resembling that of the uniform distribution.

Lastly, we note that as *n* approaches infinity, the recursive process drives the distribution toward a degenerate distribution centered at the median. Mathematically, this means that the nth level derangetropy $\rho_{f}^{\left(n\right)}\left(x\right)$ converges in distribution to a Dirac delta function δ(x−m), where all the probability mass is concentrated at the median x=m; i.e.,

(27) $$\lim_{n\to \infty }\rho_{f}^{\left(n\right)}\left(x\right)=\delta \left(x-m\right).$$

This convergence behavior is a direct consequence of the derangetropy functional’s design, which inherently favors the centralization of information. The recursive smoothing effect ensures that with each iteration, the distribution becomes more focused around the central point, ultimately leading to a situation where all mass is concentrated at the median. It is worth mentioning that the derangetropy functional exhibits self-similar patterns, particularly in the earlier stages of recursion. However, as the recursion deepens, the structure becomes more centralized about the median and less fractal-like, leading to increasingly negative scaling exponents.

## 5. Combinatorial Perspective

The concept of derangetropy is deeply rooted in combinatorial principles, particularly the notion of derangements. In combinatorics, a *derangement* is a permutation of a set where no element appears in its original position, representing a state of maximal disorder. Extending this concept to continuous probability distributions, the derangetropy functional $\rho_{f}\left(x\right)$ quantifies the local interplay between order and disorder at each point in the distribution.

A key mathematical tool in deriving the properties of derangetropy is Euler’s reflection formula, which is given by

(28) $$\sin\left(\pi z\right)=\frac{\pi }{\Gamma (z)\Gamma (1-z)}\;\mathrm{for}\;z\notin Z,$$

where Γ(z) is the Gamma function, which is a continuous generalization of the factorial function. This formula establishes a fundamental connection between the sine function and the combinatorial structures underlying the derangetropy functional. The derangetropy functional, initially defined by Formula (1), can be reformulated using Euler’s reflection formula as follows:

(29) $$\rho \left[f\right]\left(x\right)=\left(\frac{24}{e}\right)\left(\frac{1}{\Gamma (F(x))\Gamma (1-F(x))}\right)F{\left(x\right)}^{F(x)}{\left(1-F\left(x\right)\right)}^{1-F(x)}f\left(x\right).$$

This reformulation highlights the recursive self-referential nature of the distribution, where the distribution refers to its own values at each point *x*, influencing the overall informational dynamics of the system. The terms $F{\left(x\right)}^{F(x)}$ and ${\left(1-F\left(x\right)\right)}^{1-F(x)}$ introduce a self-weighting modulation mechanism that amplifies disorder nonlinearly in regions of high probability. The use of Gamma functions Γ(F(x)) and Γ(1−F(x)) further extends this concept by adding a layer of combinatorial complexity. These functions act as continuous generalizations of derangements, quantifying the degree of disorder at each point based on arrangements relative to *x*, where Γ(F(x)) accounts for elements less than *x* and Γ(1−F(x)) for elements greater than *x*. Together, the self-weighting terms and Gamma functions establish a feedback mechanism that dynamically determines the local derangetropy, highlighting how the information content and combinatorial structure interconnect across the support of the distribution.

The system governed by the derangetropy functional continuously transitions between states of order and disorder. This dynamic conversion is modulated by the structural and modulation informational energies inherent within the distribution, reflecting the probabilistic laws that govern the system’s behavior. Equilibrium is achieved when the total informational energy $E_{\mathrm{Total}}\left(x\right)$ is minimized across the distribution, representing a balance between order and disorder.

Consequently, the derangetropy functional captures both local variability and global stability within the system. At any given point *x*, the local variability is determined by whether *x* aligns with its expected order. Aggregating these local measures provides a global assessment of the system’s informational content, which is consistent with the principles of complex systems, where global properties emerge from local interactions. This dual capability makes derangetropy a powerful tool for analyzing both micro- and macro-structures within a wide range of probabilistic systems.

## 6. Applications and Examples

In this section, we provide an example demonstrating the application of derangetropy in signal processing and biological systems. In particular, we explore the application of derangetropy in electroencephalography (EEG) analysis to identify distinctions between healthy individuals and patients with Major Depressive Disorder (MDD).

**Figure 5:**
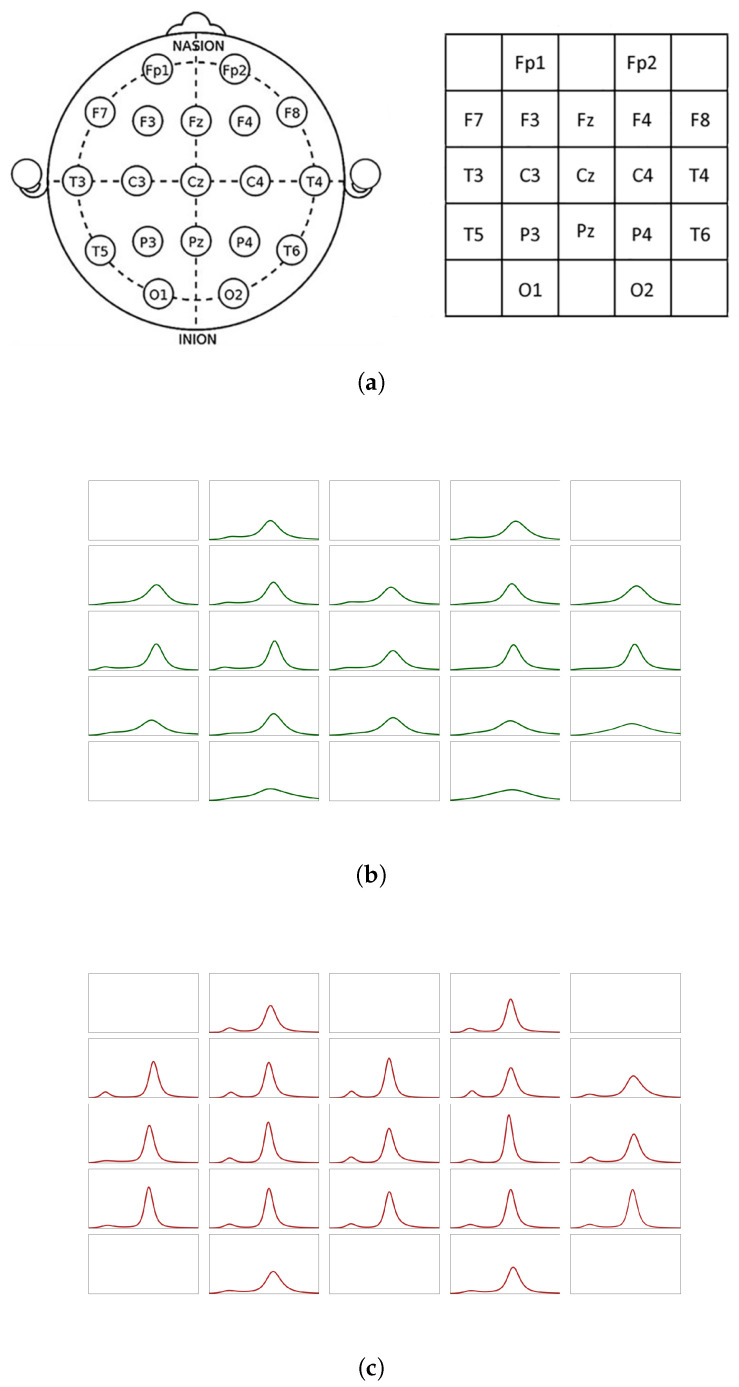
Schematics of electrode placement and derangetropy functionals for control and MDD subjects. (**a**) Electrode placement and mesh matrix representing the 10–20 international system; (**b**) plots of derangetropy functional for a healthy subject when eyes closed; (**c**) plots of derangetropy functional for a subject with MDD when eyes closed.

The EEG dataset comprises recordings from 34 patients during five-minute sessions with eyes closed, which were collected at the Hospital University Sains Malaysia (HUSM) in Kelantan, Malaysia, as documented in [11]. Using the methodology outlined in [12], we compute derangetropy functionals for action potential distributions across 19 electrode channels, comparing results from a healthy individual with those from an MDD patient, as shown in Figure 5. This analysis showcases derangetropy’s unique capacity to capture informational complexity, which surpasses the capabilities of traditional measures like differential entropy in providing meaningful insights into neural dynamics.

The differential entropy will summarize all patterns into one number. It is not clear how it should be best applied to time-series data. The derangetropy offers a more robust and generalizable framework. It not only preserves the informational structure within continuous distributions but also avoids the pitfalls associated with differential entropy’s coordinate sensitivity. This quality makes derangetropy particularly suitable for applications involving complex neural data, as it provides a coherent measure of informational complexity across different regions of the brain without the drawbacks of differential entropy.

In the frontal regions, the MDD subject exhibits higher derangetropy levels with reduced variability, indicating dysregulated but elevated neural activity, which aligns with typical patterns of emotional dysregulation observed in MDD. This finding resonates with research identifying frontal lobe abnormalities in MDD, where heightened neuronal activity is linked to impaired emotional processing and regulation [13,14]. Conversely, the healthy subject’s more variable derangetropy profile in these regions suggests a balanced and adaptive regulatory function, which is indicative of a flexible prefrontal cortex.

The temporal and parietal regions also display notable differences. For the MDD patient, lower derangetropy variability in these areas may reflect increased baseline activity, which is associated with altered sensory and emotional processing, particularly in the context of auditory and spatial perception. The literature on MDD suggests that these regions often show reduced functional connectivity and heightened sensitivity to emotional stimuli [15]. In contrast, the healthy individual exhibits more variable derangetropy, which might suggest dynamic engagement across sensory and emotional tasks consistent with typical functioning in these brain regions.

Hemispheric asymmetry is also pronounced in the MDD patient, particularly within the right hemisphere, where reduced derangetropy variability suggests sustained activation often associated with negative affect and withdrawal behaviors. This is in line with findings that connect MDD to right-lateralized neural activity patterns and emotional distress [16]. In contrast, the healthy individual displays more balanced derangetropy across both hemispheres, indicating a more flexible and adaptive cognitive state.

## 7. Conclusions and Future Work

This paper introduced derangetropy, which is a novel functional measure designed to characterize the dynamic nature of information within probability distributions. Unlike scalar measures such as Shannon entropy, derangetropy provides a functional representation, capturing the intricate interplay between order and disorder across the entire support of a distribution. Its self-referential and periodic characteristics offer a powerful framework for analyzing the stability and evolution of information, particularly in complex systems.

Building on its theoretical foundation, derangetropy has demonstrated versatility through empirical analyses of various distributions. A promising direction for future research lies in studying its temporal behavior within time-evolving systems governed by Markov chains or stochastic differential equations. These analyses could elucidate how information evolves across state transitions in Markov processes or how it transforms across multiple timescales in stochastic systems.

Derangetropy also presents significant opportunities for integration with deep neural networks. By regulating information flow during training, it has the potential to prevent overconcentration or dispersion, enhancing robustness and generalization. Furthermore, incorporating equilibrium relations into backpropagation could lead to the development of novel, more efficient optimization algorithms, particularly for high-dimensional and non-convex problems.

In summary, derangetropy represents a significant conceptual and practical advancement in information theory, providing a functional perspective to the nature of information. By extending its analysis to temporal systems and exploring its utility in optimization and predictive modeling, future research will deepen our understanding of the dynamics of information. These efforts will likely enhance the design and function of autonomous systems and offer new tools for addressing challenges in dynamical and self-organizing systems.

## Figures and Tables

**Figure A1:**
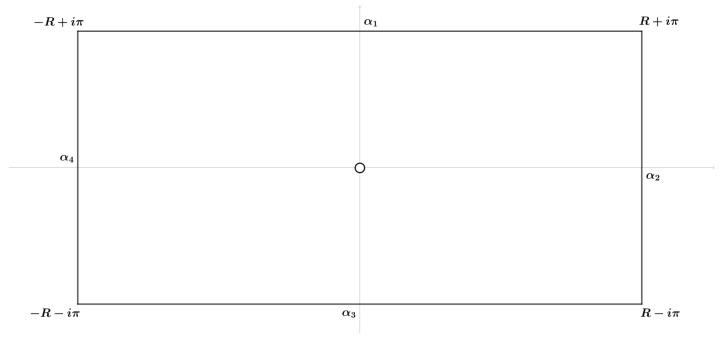
Schematic of the rectangular clockwise integration path.

## Data Availability

No new data were created or analyzed in this study. Data sharing is not applicable to this article.

## Appendix A

Let us compute $\int_{0}^{1}\sin\left(\pi z\right)z^{z}{\left(1-z\right)}^{1-z}dz$ by defining

(A1) $$\begin{array}{cc} S\;:=\; & \;\int_{0}^{1}z^{z}\;{\left(1-z\right)}^{1-z}\;e^{i\pi z}\;dz\\ \;=\; & \;\int_{0}^{1}\left(1-z\right)\;\frac{z^{z}}{{\left(1-z\right)}^{z}}\;e^{i\pi z}\;dz\\ \;=\; & \;\int_{0}^{1}\left(1-z\right)\;\frac{e^{z\log z}}{e^{z\log(1-z)}}\;e^{i\pi z}\;dz\\ \;=\; & \;\int_{0}^{1}\left(1-z\right)\;e^{z(i\pi +\log z-\log(1-z))}\;dz\;. \end{array}$$

By substituting t=logz−log(1−z), we obtain

$$z=\frac{e^{t}}{e^{t}+1}\;,$$

which in turn yields

(A2) $$\begin{array}{cc} S\;=\; & \;\int_{-\infty +i\pi }^{+\infty +i\pi }\frac{1}{e^{t}+1}\;e^{\left(i\pi +t\right)\frac{e^{t}}{e^{t}+1}}\;\frac{e^{t}}{{\left(e^{t}+1\right)}^{2}}\;dt\\ \;=\; & \;\int_{-\infty +i\pi }^{+\infty +i\pi }e^{\frac{te^{t}}{e^{t}-1}}\;\frac{e^{t}}{{\left(e^{t}-1\right)}^{3}}\;dt\;. \end{array}$$

Now, consider the following function

(A3) $$g\left(u\right)=e^{\frac{ue^{u}}{e^{u}-1}}\;\frac{e^{u}}{{\left(e^{u}-1\right)}^{3}}\;.$$

This function is meromorphic on the strip

D={u∈C:−π≤ℑ(u)≤π}

and its only point is located at u=0 having

$$Res\left(g,0\right)=-\frac{e}{24}\;.$$

Next, consider the rectangular clockwise path α, as depicted in Figure A1, such that $\alpha =\alpha_{1}⊕\alpha_{2}⊕\alpha_{3}⊕\alpha_{4}$, implying that

(A4) $$\oint_{\alpha }g\left(u\right)du\;=\;\;-2\pi i\;Res\left(g,0\right)\;=\;\;\;2i\;\frac{\pi e}{24}\;.$$

It is noted that for any sequence $\{u_{n}\}\subset D$, we have $|u_{n}|\to \infty$. In turn, this leads $g(u_{n})$ to approach infinity, implying that $\int_{\alpha_{2}}g\left(u\right)du$ and $\int_{\alpha_{4}}g\left(u\right)du$ both become zero as R→∞. By further noting that $\int_{\alpha_{1}}g\left(u\right)du=\int_{\alpha_{3}}g\left(u\right)du$, one obtains $S=\int_{\alpha_{1}}g\left(u\right)du$. Thus,

(A5) $$\int_{0}^{1}\sin\left(\pi z\right)z^{z}{\left(1-z\right)}^{1-z}dz\;=\;\frac{\pi e}{24}.$$
